# Obesity as a Risk Factor for Alzheimer’s Disease: Implication of Leptin and Glutamate

**DOI:** 10.3389/fnins.2019.00508

**Published:** 2019-05-22

**Authors:** Ana Lloret, Paloma Monllor, Daniel Esteve, Ana Cervera-Ferri, Maria-Angeles Lloret

**Affiliations:** ^1^Department of Physiology, Faculty of Medicine, University of Valencia, Health Research Institute INCLIVA, Valencia, Spain; ^2^Department of Human Anatomy and Embryology, Faculty of Medicine, University of Valencia, Valencia, Spain; ^3^Department of Clinic Neurophysiology, University Clinic Hospital of Valencia, Valencia, Spain

**Keywords:** leptin-resistance, dementia, overweight, excitotoxicity, LTP

## Abstract

Obesity is known to induce leptin and insulin resistance. Leptin is a peptide hormone synthesized in adipose tissue that mainly regulates food intake. It has been shown that insulin stimulates the production of leptin when adipocytes are exposed to glucose to encourage satiety; while leptin, via a negative feedback, decreases the insulin release and enhances tissue sensitivity to it, leading to glucose uptake for energy utilization or storage. Therefore, resistance to insulin is closely related to leptin resistance. Obesity in middle age has also been related to Alzheimer’s disease (AD). In recent years, the relation between impaired leptin signaling pathway and the onset of AD has been studied. In all this context the role of the blood brain barrier (BBB) is crucial. Slow excitotoxicity happens in AD due to an excess of the neurotransmitter glutamate. Since leptin has been shown to regulate *N*-methyl-D-aspartate (NMDA) receptors, we want to review the link between these pathological pathways, and how they are affected by other AD triggering factors and its role in the onset of AD.

## Introduction

In the last years obesity has changed from a mere aesthetic problem to become into a serious health problem worldwide. Nowadays it is considered by medical authorities as a genuine epidemic, consuming enormous technical, human, and economic resources. Obesity and also overweight affect near than 300 million people from child to elderly, and it is not related with the development level of the country (World Health Organization [WHO], 2003). Moreover, research has shown that obese children are more likely to be overweight or obese as adults (Sahoo et al., 2015). The growing incidence caused by a change in eating habits, by an increased consumption of fat and also by a substantial reduction in physical activity. This nutritional disorder implicates a number of conditions associated with excess weight, such as heart diseases, type 2 diabetes, high blood pressure, different types of cancer, and even neurodegeneration (Friedemann et al., 2012; Odegaard and Chawla, 2013; Hotamisligil, 2017). All these chronic pathologies associated with obesity, englobe the main causes of death and also monopolize 80% of healthcare expense (The World Health Organization [WHO], 2019).

The body mass index (BMI) is the most widely used method to classify a person in relation to his/her weight. In adults a BMI of 18.5 to 24.9 stands for a healthy, normal weight, while a value between 25 and 29.9 means is considered overweight. From values from 30 to 39.9 implies you are obese and from 40 to above means you are severely obese. A BMI lower than 18.5 is considered underweight and may indicate an eating disorder or malnutrition. However, BMI is not representative of overweight in the case of people with high percentage of muscle mass. In these population a high BMI would not indicate excess of fat. Perhaps a more accurate method to assess excess fat is waist circumference, which can be used as an additional measure in people who are overweight or moderately obese. Usually, men with a waist circumference of 94 cm (37 in) or more and women with a waist circumference of 80 cm (31.5 in) or more are in risk of obesity-related diseases.

In the last 15 years, obesity and dementia risk have been related (Whitmer et al., 2005). An increase in adipose tissue could promote a decrease in the blood flow to the brain, leading to vascular injury. In fact, obesity is related to changes in cerebral vascularization, because perivascular adipose tissue is not found around the cerebral arteries (Dorrance et al., 2014). A decrease in blood flow to the brain causes ischemia in vulnerable brain areas. The most sensitive areas, specifically vulnerable, are neurons located in the hippocampal regions CA1, CA3, and CA4, portions of the caudate nucleus, cerebellum, and layers III, V, VI of the neocortex (Payabvash et al., 2011). The hippocampal areas, due to its high baseline metabolic activity, are extremely susceptible to reduced oxygen and glucose intake and it is believed that it can be one of the causes of increased memory loss (Kivipelto et al., 2005). Chronic peripheral inflammation caused by the release of adipokines as leptin and other cytokines, may spread to the brain and the neuroinflammation is linked to a decrease in the brain white matter, leading to impair neuronal connections (Arnoldussen et al., 2014; Kiliaan et al., 2014). Moreover, neuroinflammation could be triggered by an imbalance in the gut microbiota due to the consumption of diets high in fats and sugars (Solas et al., 2017), which could provoke an alteration in the “gut-brain axis.”

In this review, we are going to discuss the role of the cytokine leptin in brain function and specially in the memory decline associated with Alzheimer’s disease (AD).

## Leptin and Its Role in the Brain

Leptin was discovered in Zhang et al. (1994) by Friedman and co-workers using modern molecular biology tools such as positional cloning. After cloning the *ob* gene in mice and its homolog in humans, the gene product was purified and called leptin (Maffei et al., 1995). Leptin is a hormone mainly produced by adipose tissue which is released to the bloodstream and circulates throughout the body proportionally to the body fat mass (Friedman and Halaas, 1998). Moreover, leptin is expressed either in subcutaneous and visceral adipose tissue (Lieb et al., 2009), and also in placenta, skeletal muscle, ovaries, mammary epithelial cells, (Margetic et al., 2002), or even in the gastrointestinal tract with both endocrine and exocrine actions (Cammisotto et al., 2005). Leptin can be found into the bloodstream either associated to binding proteins or in a free, bioactive form (Sinha et al., 1996). Obese individuals show a higher proportion of the free circulating leptin form and in contrast, in lean subjects leptin circulates mainly bound to its soluble receptor (Sinha et al., 1996). This is in line with the fact that one of the functions attributed to leptin is to regulate food intake and energy expenditure. When adipose tissue decreases plasma leptin levels also decrease, and when adipose tissue increases leptin levels increase and suppresses appetite (Maffei et al., 1995). But we know today that the functions of leptin are many others: it is a growth factor, a permissive factor for puberty, controls metabolism and immune system and is also implicated in memory (Margetic et al., 2002; Kelesidis et al., 2010; McGregor and Harvey, 2018a). All these effects are mediated by binding to specific leptin receptors (LepR) expressed in the central nervous system (CNS) as well as in peripheral tissues.

The LepR has six different isoforms: five of them (LepRa, LepRc, LepRd, LepRf, and LepRb) show transmembrane domain, whereas LepRe only presents an extracellular domain and acts as a soluble receptor. LepRb is the long form of the receptor while the others isoforms are shorter (Chua et al., 1997; Tartaglia, 1997; Cui et al., 2017). LepRs are widely expressed all along the body, but focusing in the brain, both short and long isoforms are broadly expressed. LepR is found in the hypothalamus (specifically, in the arcuate, ventromedial, paraventricular, and ventral premammillary nuclei) but LepRs are also present in other areas primarily non-associated with energy balance such as the neocortex, hippocampus, thalamus, leptomeninges, choroid plexus (Mercer et al., 1996; Fei et al., 1997; De Matteis and Cinti, 1998), entorhinal cortex, amygdala, and rostral medulla (Savioz et al., 1997; Burguera et al., 2000).

## Leptin and Obesity

To reach the CNS, leptin crosses the blood-brain barrier (BBB) through a saturable transport system (Banks et al., 1996). Brain microvessels express short leptin receptors which bind and internalize leptin (Karlsson et al., 1997; Bjørbaek et al., 1998). It has been proposed that leptin enters via cerebrospinal fluid (CSF) from plasma because the choroid plexus contains many leptin receptors (Schwartz et al., 1996; Golden et al., 1997). In the hypothalamus, a very specific type of cell, the tanycyte has a remarkable role conducting leptin. Tanycytes are ependymal cells located in the third ventricle and also in the floor of the fourth ventricle. They have cellular extensions that communicate deep into the hypothalamus, and thank to these cellular prolongations, the leptin is conducted to its target areas through transcytosis (Balland et al., 2014). When leptin binds to its receptor it activates several signaling cascades such as the Janus tyrosine kinase 2 (JAK2), the signal transducer activator of transcription 3 (STAT3), the phosphatidylinositol 3-kinase (PI3 kinase), and the AKT pathways (Flak and Myers, 2016) that culminates in the modification of neurons releasing three hormone-derived peptides: neuropeptide Y (NPY), pro-opiomelanocortin (POMC), and agouti-related peptide (AgRP). If the amount of NPY and AgRP, are increased, it leads to increased food intake but the activation of the POMC triggers factors (mainly the α-melanocyte–stimulating hormone) that inhibit food intake (Varela and Horvath, 2012). The administration of leptin increases POMC mRNA expression, and inhibits NPY and AgRP mRNAs translation (Elias et al., 1999; Balland and Cowley, 2015).

Nevertheless, we have a hormone that is central in the regulation of food intake and glucose levels control, and of course, we are referring to insulin. An increase in the level of circulating insulin produced by its prandial release from endogenous stores is associated with the state of satiety. Given this fact, the relation of leptin and insulin is a point to discuss. As, Kahn and Flier (2000) propose, leptin has an insulin-sensitizing effect after both an acute or a chronic administration. This could happen because LepRs are present in pancreatic β-cells (Pallett et al., 1997; Amitani et al., 2013) and when leptin binds to them, it is able to inhibit insulin synthesis and release to the bloodstream. On the contrary, insulin stimulates leptin secretion from adipose tissue closing the feedback loop. In this line, Kulkarni et al. (1997) show that leptin administration lowers insulin secretion *in vivo* not only in mice but also in isolated human islets. Making tissues more sensitive to insulin, leptin causes glucose uptake for energy utilization, or storage.

Given that insulin resistance happens in obesity, the role of leptin in obesity etiology and pathophysiology is worth to discuss. In obese subjects, levels of leptin increase in plasma compared to lean subjects. This is, probably, a physiological response to reduce food intake, and also aims to use all the energy derived from the lipid metabolism (Myers et al., 2010). However, what actually happens is that obese subjects show resistance to leptin actions over time. In this line, leptin resistance is associated with both increased circulating levels of leptin and also with inability of exogenous leptin to decrease body fat or food intake (Myers, 2015). Actually, augmented circulating levels of leptin in obesity caused hypothalamic leptin resistance, reducing the anorexigenic and energy expenditure signals and aggravating obesity (Waterson and Horvath, 2015). The cause of leptin resistance is not well elucidated, but it seems to have its origin in a defect in the transport of leptin across the BBB, maybe in deficits involving intracellular signaling mechanisms downstream of leptin receptor or even in development alterations (Banks, 2004; Myers et al., 2008; Farr et al., 2015).

## Obesity and Risk of Alzheimer’s Disease

Alzheimer’s disease (AD) is the most common form of dementia. It is characterized by two main lesions in the brain: senile plaques, predominantly formed by amyloid-beta (Aβ) peptide and neurofibrillary tangles, mainly compound by hyperphosphorylated tau protein (p-tau). Aβ is product of the processed of the amyloid precursor protein (APP) by β- and γ-secretase enzymes (Mattson, 2004; Patterson et al., 2008). The first symptom of the disease is episodic memory loss associated with hippocampal affectation. But some pathological modifications related to obesity, such as neuroinflammation, insulin resistance, or mitochondrial dysfunction, also occur in AD pathological progression (O’Brien et al., 2017).

The number of studies relating an increase in fat body mass and the risk of suffer AD has increased in the last years. However, the results are controversial and many times inconclusive. It seems that it is important to differentiate between mid-life and late-life overweight (Xu et al., 2011). Specifically, obesity in midlife and a weight loss in the preclinical phase characterizes dementia (Singh-Manoux et al., 2018). In fact, in a recent meta-analysis of 21 studies, the authors conclude that obesity below the age of 65 years (midlife obesity) correlates with the incident of dementia, but not the late-life obesity (over 65 years) (Pedditizi et al., 2016). Very recently, Kivimäki et al. (2018) analyzed 1,349,857 people from 39 different cohorts with BMI data assessed at baseline. The authors find that 20 years before dementia diagnosis, higher BMI is associated with increased dementia risk in mid-life. Moreover, they describe that this risk is reversed in late-life and a higher BMI could even be protective (Kivimäki et al., 2018).

Furthermore, a meta-analysis of 15 prospective studies including more than 72000 participants used BMI measures and the authors found that both underweight and obese are related to an increase risk of AD but only in mid-life; high BMI in late-life was not associated with any dementia (Anstey et al., 2011). Moreover, the authors conclude that underweight could be a useful marker for identifying mild cognitive impairment (MCI) subjects at increased risk to convert to AD (Joo et al., 2018). Another very large retrospective cohort study with two million people analyzed, concludes that underweight in both middle and old age increases the risk of dementia over two decades (Qizilbash et al., 2015), although this study is not focused specifically in AD. In spite of these publications, the hypothesis that being overweight in mid-life is linked to dementia in late-life seems to be widely accepted by scientific community. A recent analysis explains that the duration of the preclinical weight loss phase could be a negative confounding parameter and a plausible explanation of this paradox (Pegueroles et al., 2018).

## The Role of Leptin in AD

Since obesity and dementia were related, many studies tried to find a link between brain leptin activity and AD development. In this line, Bonda et al. (2014) show that leptin is increased in the CSF and also in the hippocampus of AD patients, but leptin receptor mRNA is decreased within degenerating neurons and this could suggest a novel neuronal leptin resistance in AD. On the other hand, Maioli et al. (2015) show no changes in leptin concentration in CSF, but LepR also diminishes in post-mortem brains of AD patients, confirming that leptin resistance occurs. LepR decreased expression related to age, is also shown in an animal model of AD (King et al., 2018).

Brain leptin resistance is proposed as part of the neurodegenerative process. Leptin has both neurotrophic and neuroprotective properties therefore, leptin signaling deficits may lead to susceptibility to AD-related neurotoxic conditions. In fact, leptin is able to modify the levels of Aβ peptide by limiting its production in neurons via reducing β-secretase activity (Fewlass et al., 2004; Marwarha et al., 2010). Likewise, leptin protects hippocampal neurons in primary cell culture from Aβ derived insults such as oxidative stress (Martins et al., 2013). Moreover, leptin enhances the removal of Aβ by promoting its clearance and degradation and activating the insulin degrading enzyme (Patterson et al., 2008). Furthermore, in neurons treated with Aβ, leptin prevents glycogen synthase kinase 3β (GSK3β) activation (Greco et al., 2009; Marwarha et al., 2010; Martins et al., 2013). This is very significant for AD pathogenesis since GSK3β is a kinase of tau and is implicated in the formation of neurofibrillary tangles. Besides, development of leptin resistance is linked with higher tau pathology in transgenic mouse models of AD suggesting that a defect in LepR-mediated signaling cascade could increase p-tau levels (Platt et al., 2016).

An important target of leptin action is the hippocampus, where it has a role in synaptic plasticity process, in memory preservation, and has pro-cognitive effects (Harvey, 2007, 2013). All these effects seem to be mediated by modulating glutamate receptors: the ionotropic α-amino-3-hydroxy-5-methyl-4- isoxazolepropionic acid (AMPA) and *N*-methyl-D-aspartate (NMDA). These receptors are involved in long-term potentiation (LTP) and in long-term depression (LTD). Leptin enhances LTP and decreases LTD, increasing the efficacy of excitatory synaptic transmission (Shanley et al., 2001; Wayner et al., 2004; Moult and Harvey, 2011; McGregor and Harvey, 2018b; McGregor et al., 2018). Moreover, leptin resistance is determinant for hippocampal dysfunction (Mainardi et al., 2017). In AD models, leptin prevents the anomalous effects of Aβ on hippocampal LTP and LTD, restoring normal hippocampal synaptic function (Doherty et al., 2013), and also increasing the synaptic density and rescuing memory deficits (Perez-Gonzalez et al., 2014).

**FIGURE 1:**
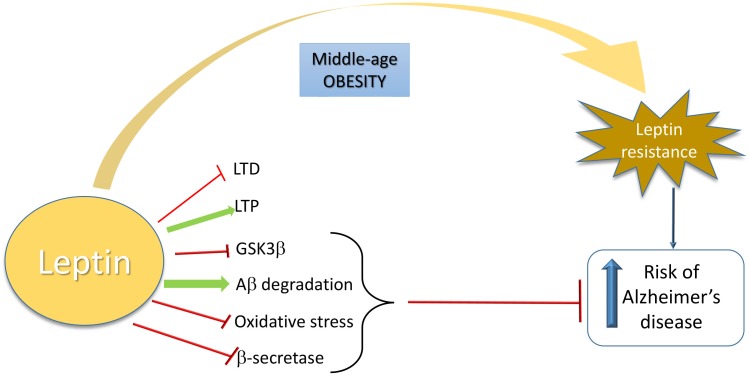
Schematic overview of the relationship between leptin and increased risk of Alzheimer’s Disease. Leptin is neuroprotective in AD by inhibiting LTD, GSK3β, oxidative stress, and β-secretase activity and by inducing LTP and Aβ degradation. When a leptin-resistance takes place due to middle-age obesity, AD risk is increased.

Taken together, the studies described above indicate that brain leptin resistance could be central in AD pathophysiology, including the regulation of glutamatergic connections involved in hippocampal LTP and LTD. A schematic view is shown in Figure 1.

## Glutamate, Obesity and AD Are Linked Via Leptin-Resistance

Mild cognitive impairment and AD patients show an increase in plasma glutamate and glutamine (Miulli et al., 1993; Trushina et al., 2013). This increment is also reflected in brain, since some studies identify an increase in glutamate and glutamine levels in CSF from AD (Pomara et al., 1992; Jimenez-Jimenez et al., 1998; Kaiser et al., 2010; Madeira et al., 2018) and from MCI patients (D’Aniello et al., 2005). If this increase comes directly from the arise in the peripheral levels of glutamate and glutamine or if it is an indirect phenomenon is not yet known in AD. Curiously, glutamine levels increase in hippocampus from mice fed with a high-fat diet during 6 months (Lizarbe et al., 2019). Moreover, the BBB is disrupted in early phases of the disease and the consequences of this disruption in the amino acid transport are not yet studied in depth (Montagne et al., 2017). In any case, a slow excitotoxicity is shown in AD and this consist of an overexcitation of NMDA receptors by glutamate (Beal, 1992; Ong et al., 2013). Glutamate overexcite the NMDA receptors in a tonic manner and a good evidence of this, is that memantine, an uncompetitive NMDA receptor antagonist, is a well-established treatment of AD (Parsons et al., 2007). In fact, Aβ causes the increase of glutamate (Fuchsberger et al., 2016) and the intraneuronal Ca^2+^ levels (Kuchibhotla et al., 2008). A pathological signaling cascade is triggered, involving an increase of Cdk5-p35 levels, a decrease of Cdh1 and finally glutaminase increase, causing a positive feedback loop of excitotoxicity (Fuchsberger et al., 2016). Interestingly, Cdk5-p35 also modulates signaling induced by leptin (He et al., 2009). Cdk5-p35 causes SOCS3 activation, a negative feedback regulator which inhibits leptin-induced signal transduction and causes leptin resistance (He et al., 2009). So, the excess of glutamate levels can cause a cascade of events that also induce leptin-resistance.

**FIGURE 2:**
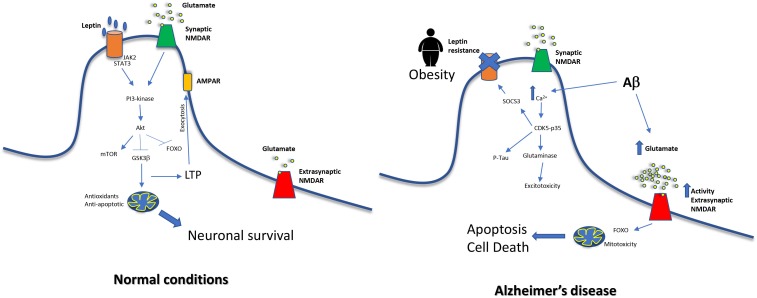
Relationship between middle-age obesity, glutamate excitotoxicity and increased risk of Alzheimer’s disease. In normal conditions, NMDA signaling and leptin receptor signal merge, exerting neuroprotective effects on cells. On the other hand, leptin resistance impairs this pathway. Moreover, Aβ causes an increase of glutamate levels and this leads to a dysfunction in extrasynaptic NMDAR, a decrease in LTP and mitochondrial alterations.

Interestingly, in AD the aforementioned overactivation is produced in extrasynaptic NMDA receptors rather than in synaptic NMDA receptors (Zhang et al., 2016). Overstimulation of synaptic NMDA receptors is considered neuroprotective and in contrast, the overstimulation of extrasynaptic NMDA receptors induces tau hyperphosphorylation (Sun et al., 2016) and cell death (Hardingham and Bading, 2010). In fact, memantine blocks preferentially extrasynaptic over synaptic NMDA receptors (Xia et al., 2010) as part of its action as AD treatment. NR2-A is a subunit mainly present in synaptic NMDA receptors and it has shown that leptin mediates neuroprotection activating them (O’Malley et al., 2007), and this is critical for the induction of LTP and LTD (Muller et al., 2009). When leptin binds to its receptor, activates JAK2, which in turn promotes the activation of STAT3, and then, PI3K-Akt signaling pathways are induced. Activation of synaptic NR2A-containing NMDARs by glutamate also induces the PI3K-dependent pathway (Lee et al., 2002), so both common signals are highly potentiated. The signal cascade will induce AMPA exocytosis and LTP (Moult et al., 2010) but also neuronal survival by promoting expression of mitochondrial antioxidant enzymes and anti-apoptotic proteins such as Bcl-xl (Guo et al., 2008), and by inhibiting Foxo (Al-Mubarak et al., 2009) and GSK3β (Greco et al., 2009). In contrast, in AD extrasynaptic NMDA receptors are overstimulated and this leads to neuronal death. Extrasynaptic NMDAR induces the pro-apoptotic transcription factor Foxo (Dick and Bading, 2010) and also mitotoxicity. The consequences are mitochondrial calcium sustained increase, compromised ATP production and mitochondrial dysregulation finally inducing cell death (Bading, 2017). A global scheme is shown in Figure 2.

## Conclusion

Leptin is a hormone secreted by adipose tissue that matters for the correct functioning of the brain, including the memory, and learning processes in the hippocampus. Leptin is neuroprotective and increase LTP, potentiating the activity of synaptic NMDA receptors of glutamate. We discussed how in AD both leptin resistance, LTP dysfunction, and also an increase in glutamate happen. For all this, obesity in middle-age could be considered as a risk factor to develop AD in the elderly.

## Author Contributions

All authors listed have made a substantial, direct and intellectual contribution to the work, and approved it for publication.

## Conflict of Interest Statement

The authors declare that the research was conducted in the absence of any commercial or financial relationships that could be construed as a potential conflict of interest.
